# Oncology nurses’ perceptions of obstacles and role at the end-of-life care: cross sectional survey

**DOI:** 10.1186/s12904-017-0257-1

**Published:** 2017-12-19

**Authors:** Aurelija Blaževičienė, Jamesetta A. Newland, Vilija Čivinskienė, Renea L. Beckstrand

**Affiliations:** 1 Department of Nursing and Care, Medical Academy, Lithuanian University of Health Sciences, Kaunas, Lithuania; 2 New York University Rory Meyers College of Nursing & NYU Langone Health Ambulatory Care West Side, New York, NY 10010 USA; 3 Brigham Young University College of Nursing, Provo, UT 84602 USA; 4 Head of Nursing and Care Department Faculty of Nursing, Medical Academy Lithuanian University of Health Sciences, Jankaus st.4, LT - 44307 Kaunas, Lithuania

**Keywords:** Oncology nurses, End-of-life care, Obstacles to end-of-life care, Nurse role, Supporter, Advocate, Information broker

## Abstract

**Background:**

Major obstacles exist in the care of patients at the end of life: lack of time, poor or inadequate communication, and lack of knowledge in providing care. Three possible nursing roles in care decision-making were investigated: Information Broker, Supporter, and Advocate. The purpose of this study was to examine obstacles faced by oncology nurses in providing end-of-life (EOL) care and to examine roles of nurses in providing care.

**Methods:**

A descriptive, cross-sectional, correlational design was applied. The study was conducted at two major University Hospitals of Oncology in Lithuania that have a combined total of 2365 beds. The study sample consisted of 239 oncology registered nurses. Data collection tool included a questionnaire about assessment of obstacles and supportive behaviors, nursing roles, and socio-demographic characteristics.

**Results:**

The two items perceived by respondents as the most intense obstacles to providing EOL care were The nurse’s opinion on immediate patient care is not welcome, valued or discussed and.

Family has no access to psychological help after being informed about the patient’s diagnosis. The majority of respondents self-assigned the role of Supporter.

**Conclusions:**

Major obstacles in providing care included the nurse’s opinion that immediate patient care was not valued, lack of nursing knowledge on how to treat the patient’s grieving family, and physicians who avoided conversations with the patient and family members about diagnoses and prospects. In EOL care nurses most frequently acted as Supporters and less frequently as Advocates.

## Background

According to the World Health Organization (WHO), worldwide 56.2 million people die every year. Of these, 7.6 million people die from cancer. In Europe, 3.2 million people die each year and 1.7 million deaths are caused by cancer [1]. Patients spend a significant period of time in oncology hospitals where primarily nurses are responsible for end-of-life (EOL) care. Throughout history nurses have been responsible for ensuring the quality of life for patients, their families, and the community through all stages of life [2]. Nurses spend more time with patients than any other health care professionals [3, 4]. Nurses provide regular care for patients at the EOL; they may identify behaviors that obstruct or improve EOL care for patients and families [5]. Furthermore, identifying the obstacles or supportive behaviors that have the most impact to patients and families and then working to eliminate highly rated obstacles or increase support for positive behaviors are critical to improve EOL care.

Research findings have indicated that the main obstacles in caring for patients at the EOL include the lack of time for professional care and staff shortages; challenges in communication with colleagues, patients, and/or patients’ relatives; intensive treatment decisions made in spite of patients’ wishes and needs, and a lack of knowledge about care for patients at the EOL [6]. Of 77 articles published in the last 10 years on obstacles to EOL care in intensive care units, palliative care units, and oncology hospitals only a few studies analyzed nursing roles and obstacles faced by nurses [2, 7].

In the *Medical Dictionary of Health Terms*, the “end-of-life” concept refers to a final period – hours, days, weeks, or months in a person’s life in which it is medically obvious that death is imminent or a terminal moribund state cannot be prevented [8]. Similarly, Watson et al. defined “end-of-life care” as the delivery of care during the last few weeks of life and the time directly preceding death in emergency departments [9]. Care of patients at the EOL involves many aspects: pain and symptom management, dealing with culturally sensitive issues, support for patients and their families during the process of dying and experiencing loss, and ethical decision-making. A survey of relevant literature revealed there were obstacles preventing nurses from demonstrating their professional competencies in EOL care [10].

Lack of time remained one of the major obstacles. Nurses had too many tasks to take time to listen to patients’ wishes concerning EOL decisions, to communicate with families; and to understand their values, expectations, and attitudes [11]. Even though nurses knew that their presence at a patient’s bed would reassure and comfort the dying person, they had no time to do that because of responsibilities for other patients too [3]. Another obstacle identified in the literature was poor or inadequate communication. Anselm et al. found that physicians, residents, and nurses reported that the main obstacles in communication with patients at the EOL were the patients themselves, the health care system, health care providers, and the nature of this dialogue [12]. Findings from Heyland et al. demonstrated that for patients high-priority communication areas that needed improvement were related to feelings of peace, assessment and treatment of emotional problems, physician availability; and satisfaction that the physician took a personal interest in them, communicated clearly and consistently, and listened. Similar areas were identified by family members as high in priority [13].

Several studies identified the lack of knowledge about care for patients at the EOL as an obstacle [6, 7, 14]; the lack of skills as well as how to treat the grieving family was a major obstacle in providing quality care. Reinke et al. analyzed what nursing skills were important but under-utilized in EOL care. Nurses named as very important such skills as communication; symptom management competencies, especially those concerning anxiety and depression; and interactions with patient-centered care systems [10].

An analysis of obstacles in EOL care must take into account the role assumed by nurses because they are placed in a unique position; they may assist patients and family members by providing information, discussing and advocating for patients’ wishes [15]. Adams et al. revealed that usually three possible nursing roles existed in EOL decision-making, namely: Information Broker, Supporter; and Advocate [16]. Still, there is little evidence on nurses’ roles in EOL decision-making. As Information Brokers nurses played an important role in the process of ensuring smooth communication between family members and the team of health care professionals. Nurses provided information to physicians and family members and also acted as mediators. Liaschenko et al. further defined the nurse’s role as the main point for exchange of information: obtaining information from many sources, synthesizing it, and forming a holistic assessment [17]. Another EOL nursing role was that of a Supporter. Nurses expanded their role in the EOL decision-making process by demonstrating empathy for patients, family members, and physicians; acting as supporters at the patient’s EOL period and developing trusting relationships with family members [15, 16]. Finally, the most researched nursing role in EOL care was that of an Advocate. This role was performed through speaking to the team of health care professionals on behalf of the patient or family as well as speaking to the family on behalf of the patient [5, 7].

Thus, defined nursing roles and activity areas at the patient’s EOL help in ensuring that unique needs are met for patients at EOL. The role of a nurse in providing EOL care is very complex, requiring personal psychological preparations, flexibility, and strength. Citation?

No such studies existed in either Lithuania or any other Eastern European country; therefore, this was the first study to investigate whether Eastern European countries faced the same challenges in EOL care as Western European countries and the United States (US), and what was the role of nurses.

### Aim

The main purpose of this study was to examine obstacles faced by oncology nurses in providing EOL care. A secondary purpose was to examine roles of nurses in providing EOL care.

## Methods

### Research design

A descriptive, cross-sectional, correlational design was applied in this study.

### Instruments

Pre-established obstacles and supportive behaviors on the questionnaire administered in this study were from an original validated survey, *Questionnaire of Helps and Obstacles in Providing End-of-Life Care to Dying Patients and Their Families* [14] that was modified after expert opinion and suggestions from oncology nurses in order to be most influential in the oncology setting. A translated and validated Lithuanian version following the technique of inverse translation was used in this study. The questionnaire contained 67 items consisting of three parts: assessment of obstacles and supportive behaviors, nursing roles, and socio-demographic characteristics. The first 40 items evaluated obstacles and supportive behaviors. Responses were given in a Likert scale format ranging from 1 = not help (or not an obstacle) to 5 = extremely intense help (or extremely large obstacles).

Items 41 to 59 evaluated the nurse’s opinion about their roles during EOL care. Nine items (41, 42, 43, 44, 45, 53, 56, 58, 59) described the role of a nurse as a Supporter; four items (48, 50, 51, 57) described the role of a nurse as an Information Broker; and six items (46, 47, 49, 52, 54, 55) evaluated the role of a nurse as an Advocate. According to a 5-point Likert scale, respondents had to evaluate different statements by marking 1 of 5 possible responses: “Strongly agree”, “Agree”, “Undecided”, “Disagree”, or “Strongly disagree”. For purposes of statistical analysis, responses were collapsed into three categories: “Strongly agree” and “Agree”, “Undecided”, and “Strongly disagree” and “Disagree”. Seven questions identified socio-demographic characteristics. After completing this study, the Cronbach’s alpha established for the questionnaire used in this study was 0.86, meeting the requirement for acceptance. Similar questionnaires have been used in studies with Intensive Care Unit nurses in Spain and the US [6, 18, 19].

### Sample and setting

Registered oncology nurses from two major Lithuanian hospitals of oncology participated in this study. The two hospitals have a combined total of 2365 beds. According to data of the Health Care Ministry, at present there are 22,300 registered nurses (RNs) in Lithuania, which includes oncology nurses (approximately 350 oncology nurses). All 250 RNs working in oncology at the two largest University Hospitals of Oncology in Lithuania were invited to take part in this study. The response rate was 95.6% with 239 participants, who indicated age, gender, employment, current work place, and length of current employment on the socio-demographic section of the questionnaire. The sample included 238 females and 1 male. The average age of nurses participating in the study was 44.09 ± 8.96 years; and the average length of service was 22.90 ± 9.66 years.

### Data collection

One of the authors personally distributed the questionnaires to all 250 eligible nurses at the hospitals during work hours from 1 September to 1 November in 2015.

### Data analysis

Survey data were processed and analyzed using the statistical software package SPSS for Windows 19.0 [20]. The level of significance selected for testing data points was established at *p* ≤ 0.05. Descriptive statistics were used to calculate the average values of the variables within a 95% confidence interval. The standard deviation was used to describe the spread of values. A statistical analysis of qualitative ordinal variables was carried out by means of the chi-square (χ^2^) test, and degrees of freedom (*df*) was calculated. The Spearman’s correlation coefficient (*r*) was used to determine the degree of dependence between variables. A positive *r* value indicated a direct linear correlation, i.e., when the value of one variable increased, the value of the other variable also increased. A negative *r* value indicated a reverse correlation, i.e., when the value of one variable increased, the value of the other variable decreased. Because the number of male nurses was not representative, no analysis of results based on gender was done.

### Ethical considerations

Research was carried out in accordance to ethical principles of scientific research, the Declaration of Helsinki, as well as the Code of Ethics of the Lithuanian Social Research Centre (LSRC). Hospital administrations were informed of the research goals, and their permission was obtained prior to starting the study. In addition, verbal informed consent was obtained from each participant of the study following an explanation of the research goals. Confidentiality of respondents was assured. Anonymity was maintained, as respondents were never asked for any identifiers such as their names, surnames, or addresses. Collected data were summarized and reported in the aggregate and used only for scientific purposes. The study was approved by the Bioethics Centre at the Lithuanian University of Health Sciences (BEC-KS (M)-566). Participants were informed about the purpose of the study, the data protection rights, and the right to refuse participation in the study or to terminate the participation without reasoning or penalty. Survey methodology was applied with minimal risk or harm to study participants.

## Results

### Obstacles to providing the EOL care

The two items perceived as the most intense obstacles to providing EOL care by respondents were identified in the following statements: The nurse’s opinion on immediate patient care is not welcome, valued or discussed (*M* = 2.01, 95% CI [1.89, 2.13]) and Family has no access to psychological help after being informed about the patient’s diagnosis (*M* = 1.88, 95% CI [1.76, 2.00]). Accordingly, two main perceived potential obstacles in critically ill patients’ care were The lack of nursing knowledge on how to treat the patient’s grieving family (*M* = 1.76, 95% CI [1.64, 1.87]) and Physicians who were evasive and avoided conversation with the patient and/or family members (*M* = 1.72, 95% CI [1.60, 1.83]). Nurses indicated that the least important obstacle in the EOL care was the patient’s family (*M* = 1.20, 95% CI [1.12, 1.27]), who may have inadequate reactions and interfere with the nurse’s duties (Table 1).

**Table 1:** General assessment of nurses’ perceptions of potential obstacles to providing the end of life care

No.	Statement	Mean	Standard deviation	95% CI	N (%)^a^
1.	The nurse’s opinion on immediate patient care is not welcome, valued or discussed	2.01	0.96	1.89–2.13	137 (57.3)
2.	Family has no access to psychological help after being informed about the patient’s diagnosis	1.88	0.93	1.76–2.00	219 (91.6)
3.	The lack of nursing knowledge on how to treat the patient’s grieving family	1.76	0.90	1.64–1.87	131 (54.8)
4.	Physicians are evasive and avoid conversation with the patient and/or family members	1.72	0.89	1.60–1.83	141(58.9)
5.	Physicians are too optimistic about the patient’s survival prospects during conversations with the patient’s family members	1.65	0.81	1.55–1.75	134 (56.0)
6.	The patient’s family does not accept information provided by a physician about the patient’s poor prognosis	1.54	0.79	1.44–1.64	155 (64.8)
7.	Family members or friends regularly call for a nurse in order to find out about the patient’s condition instead of addressing an informed family member	1.54	0.78	1.44–1.63	153 (64.0)
8.	The patient’s family members disagree on what kind of care is the most adequate	1.53	0.75	1.44–1.63	149 62.3
9.	Usually there is no time for conversations with patients about their wishes concerning the end of life decisions	1.28	0.66	1.19–1.36	201 (84.0)
10.	The patient’s relatives having inadequate understanding of the situation interfere with the nurses’ duties	1.21	0.60	1.14–1.29	210 (87.8)
11.	Nurses have to deal with angry patient’s family members	1.20	0.57	1.12–1.27	219 91.6

^a^Positive perceptions (Strongly agree/Agree)

### The nurse’s role in relation to obstacles in providing EOL care

In this study, a survey of 239 oncology nurses revealed that almost half (46%) of respondents self-assigned the role of a Supporter. Subscale values were distributed from 9 to 14 for Supporter, from 6 to 11 for Advocate, and from 4 to 8 for Information Broker. The subscale means, standard deviation, and confidence intervals are presented in Table 2.

**Table 2:** Assessment of subscales according to nurses’ roles in the end of life care

Subscale	N (%)	Mean	Standard Deviation	95% CI
Supporter	109 (46**%**)	9.56	0.88	9.44–9.67
Advocate	77 (32**%**)	6.69	1.07	6.55–6.83
Information Broker	53 (22**%**)	4.63	1.04	4.49–4.76

An analysis of obstacles in accordance to nurses’ roles did not reveal any statistically significant differences. However, three major obstacles were identified by more than 80% of all categorized respondents: Nurses have to deal with angry patient’s family members; The patient’s relatives having inadequate understanding of the situation interfere with the nurses’ duties, and Usually there is no time for conversations with patients about their wishes concerning the end of life issues/decisions. The perception of whether it was an obstacle or not an obstacle was close to being evenly divided (approximately 45%) by all categorized respondents for item The nurses’ opinion on immediate patient care is not welcome, valued or discussed. (see Table 3).

**Table 3:** Assessment of nurses’ perceptions of potential obstacles to providing the end of life care based on nurses’ roles

No.	Statement		Strongly agree/ Agree	Undecided	Strongly disagree/ Disagree	χ2	*p*	*df*
			%	%	%			
1.	The nurse’s opinion on immediate patient care is not welcome, valued or discussed	Supporter	44.2	9.5	46.3	6.08	0.19	4
		Advocate	45.5	9.1	45.5			
		Information Broker	43.4	9.8	46.8			
2.	Family has no access to psychological help after being informed about the patient’s diagnosis	Supporter	49.4	12.6	38.1	4.59	0.41	4
		Advocate	50.0	12.7	37.3			
		Information Broker	49.2	12.2	38.6			
3.	The lack of nursing knowledge on EOL care and how to treat the patient’s grieving family	Supporter	54.1	15.2	30.7	2.04	0.57	4
		Advocate	54.6	15.0	30.5			
		Information Broker	56.1	16.1	27.8			
4.	Physicians are evasive and avoid conversation with the patient and/or family members	Supporter	58.8	10.8	30.3	0.55	0.89	4
		Advocate	59.5	10.5	30.0			
		Information Broker	60.5	11.2	28.3			
5.	Physicians are too optimistic about the patient’s survival prospects during conversations with the patient’s family members	Supporter	56.3	23.8	19.9	4.16	0.42	4
		Advocate	56.4	23.6	20.0			
		Information Broker	55.1	25.4	19.5			
6.	The patient’s family does not accept information provided by a physician about the patient’s poor prognosis	Supporter	64.9	16.9	18.1	3.54	0.49	4
		Advocate	63.7	17.3	19.1			
		Information Broker	63.4	18.5	18.1			
7.	Family members or friends regularly call for a nurse in order to find out about the patient’s condition instead of addressing an informed family member	Supporter	64.1	19.0	16.9	3.35	0.52	4
		Advocate	65.0	18.2	16.8			
		Information Broker	63.9	19.0	17.1			
8.	The patient’s family members disagree on what kind of care is the most adequate	Supporter	62.3	22.5	15.2	4.73	0.35	4
		Advocate	62.3	22.3	15.5			
		Information Broker	61.5	22.4	16.1			
9.	Usually there is no time for conversations with patients about their wishes concerning the end of life issues/decisions	Supporter	84.4	4.3	11.3	0.67	0.87	4
		Advocate	85.4	4.1	10.4			
		Information Broker	85.4	4.4	10.3			
10.	The patient’s relatives having inadequate understanding of the situation interfere with the nurses’ duties	Supporter	88.3	3.0	8.7	3.83	0.48	4
		Advocate	87.3	3.2	9.5			
		Information Broker	87.3	3.4	9.3			
11.	Nurses have to deal with angry patient’s family members	Supporter	88.8	3.0	8.2	2.92	0.57	4
		Advocate	89.1	2.7	8.2			
		Information Broker	88.3	2.9	8.8			

## Discussion

The main goal of nurses in solving problems at the EOL is to reduce the patient’s suffering and manage pain and symptoms so that the patient’s quality of life would remain the same [2]. Beckstrand et al. confirmed that one of the major obstacles to autonomous decision-making was the nurse’s opinion of not being valued [11]. Data from this study confirms overall research findings that oncology nurses still lack professional autonomy because they identified disregard and disrespect of their opinion on patient care as the most significant obstacle. Study data lead to an assumption that according to nurses’ opinions, their function in EOL care was to assist physicians rather than to make autonomous decisions. This approach is very characteristic of the culture in Eastern European countries where nursing science is still developing and paternalistic relations dominate in the health care system [21]. Conflicting views and feeling that you as nurses are being disrespected often cause problems.

In this study, another dominant obstacle in the provision of EOL care was related to family members who had no access to psychological help after being informed about the patient’s diagnosis. A descriptive study conducted in intensive care units in Spain also confirmed these findings and identified the lack of support for family members as one of the obstacles affecting nursing [18]. Another study by Beckstrand et al. also revealed that the inability of family members to obtain psychological help after being informed about the patient’s diagnosis was considered an important obstacle in the provision of quality EOL care [14].

As demonstrated in the research literature, this is a universal problem in the US as well as Europe where there are no uniform systems to ensure quality health care services during critical moments of life, not only for patients but also for their family members [13, 18, 22]. Professional knowledge, skills, and coordination are necessary for problem management. Furthermore, the nursing literature contains limited information about patient care at the EOL, and nurses perceive this as an obstacle in the provision of care. A study on EOL care conducted by Hebert et al. demonstrated that 71% of nurses participating in the study lacked adequate knowledge on pain management, 62% of nurses lacked general knowledge on EOL problems, and 59% of nurses rated knowledge on management of other symptoms as inadequate [2]. Findings of our study correspond to findings of other researchers because the majority of oncology nurses participating in this study indicated a lack of nursing knowledge and training on how to treat the patient’s grieving family. Other researchers had similar findings; the lack of nurses’ knowledge was considered a major nursing obstacle [14, 18].

Skill development in key aspects of care provision may improve the provision of EOL care for critical care patients and their families. Physicians who are evasive and avoid conversation with the patient and/or family members were identified as one of the most important obstacles in nursing. Sufficient information, communication, and relationships between the staff and the relatives may help to facilitate shared decision-making. Gjerberg et al. conducted a study on EOL care communications and shared decision-making in Norwegian nursing homes; most relatives expressed that they wanted a conversation about the patient’s wants and preferences for EOL care, even when such conversations might be emotionally difficult [22]. This is also confirmed by a qualitative study carried out by the US researchers. Oncology nurses participating in the study identified subject areas they found difficult to discuss with the EOL patients. These include dialectic tensions, specific EOL related topics, the lack of skills in providing empathy care, characteristics of patients and their family members, and institutional obstacles [23].

Findings by Beckstrand et al. and Iglesias et al. correspond to this study’s results and identify physicians avoiding conversations with the patient and family members as one of the most important obstacles in the provision of EOL care [14, 18].

According to study results, an analysis of obstacles in accordance to nurses’ roles identified the following three major obstacles: Nurses have to deal with angry patient’s family members; The patient’s relatives having inadequate understanding of the situation interfere with the nurses’ duties; and Usually there is no time for conversations with patients about their wishes concerning the end of life decisions. These obstacles were described as very important in other studies conducted in both oncology and intensive care units [11, 24, 25]. Similar results were found in a study by Kirchhoff et al. One of the major obstacles in the provision of nursing care was related to issues with patients’ families that made care at the EOL more difficult, such as the family not fully understanding the meaning of life support and angry family members. A study by Beckstrand et al. had similar findings. One dominant obstacle was patients’ family members’ not understanding what the term “lifesaving measures” really meant [14].

Most frequently, nurses acted as Advocates on behalf of patients and family members by informing physicians about patients’ wishes and speaking with physicians for the family. Results of more recent studies have shown that nurses were more likely to employ direct methods, i.e., to speak with physicians and family members about the patient’s prospects and involvement in decision-making [26]. Bach et al. assessed the nurses’ role in EOL decision-making in a critical care unit. Research data revealed that nurses usually assumed the role of a Supporter – “supporting to journey” - being there, a voice to speak up, enabling coming to terms, and helping to let go [27]. Swedish researchers also analyzed which nursing role was the most frequent in an intensive care unit. According to findings of that study, nurses most frequently acted as Supporters: uncertainty about who was the close relative, getting near, keeping hope alive, and being honest, and in certain situations being Advocates [28].

Summarizing all research findings and comparing them to this study’s results, it may be argued that in EOL care nurses most frequently act as Supporters, less frequently as Advocates and even less frequently as Information Brokers. Patients and family members need assistance and support in making difficult EOL decisions, and the nurse is the person who spends the most time with the patient and family members. For this reason, support is sought among nurses as they are empathic and represent the interest of both the patient and his family. Nursing professionals are in key positions to support EOL decisions and to advocate for patients and families across all health care settings. Support and advocacy have been identified as the common thread of quality EOL nursing care [5].

### Relevance to clinical practice and education

Oncology nurses are professional and have sufficient skills and experience to play an important role in solving patients’ problems at the EOL period. Recommendations to hospital administration include providing support to oncology nurses, including strategies that would help improve the authority of the nursing profession. They should also create a physical environment in which nurse area able to talk with family about EOL issue. In addition, the information regarding identified obstacles and nurses’ roles in providing EOL care can be used to facilitate discussion and change within oncology interdisciplinary teams and improve EOL care for patients with cancer and their families. Therefore, organizing interprofessional team conferences to discuss cancer patient cases and conducting patient satisfaction surveys to move toward patient-cantered care would be useful.

Moreover, nursing education programs should have more study time on the death and dying process, and the topic of “death” should not be considered taboo. Including more credits on inter-professional communication in the training programmes would enable nurses to discuss the EOL issues with patients, their family members, and colleagues.

## Conclusion

Having analyzed study results, it is possible to conclude that respondents identified the following as major obstacles in providing EOL care: the nurse’s opinion on immediate patient care was not valued, the lack of nursing knowledge on how to treat the patient’s grieving family, and physicians avoiding conversations with the patient and family members on diagnosis and prospects. In EOL care nurses most frequently act as Supporters and less frequently as Advocates. Furthermore, three major obstacles were identified throughout all nursing roles: dealing with angry patient’s family members, the lack of time for conversations with patients about their wishes and the patient’s relatives having inadequate understanding of the situation interfere with the nurses’ duties.

## Abbreviations

EOL: End of life
